# Reproductive Efficiency of Low-Fertility Dairy Cows Submitted to Presynch-Ovsynch or Double-Ovsynch Scheme for First Service

**DOI:** 10.3390/ani15040536

**Published:** 2025-02-13

**Authors:** Luis Gustavo Delamanha Mendonça, Kenneth Wronka, Guy Fridkovski, Todd Bilby, Jeffrey Stevenson

**Affiliations:** 1 Merck Animal Health Technology Labs, Rahway, NJ 07065, USA; 2 Merck Animal Health, Rahway, NJ 07065, USA; 3 MSD Animal Health Technology Labs, Netanya 4250553, Israel; 4 Department of Animal Sciences and Industry, Kansas State University, Manhattan, KS 66506, USA

**Keywords:** culling, Fertility Index, pregnancy per AI

## Abstract

Reproductive efficiency has a major impact on the profitability of dairy operations. Focusing on improving the reproductive performance of cows with expected reduced fertility is desired; however, further research is needed in this area. The objectives of this study were to identify a subpopulation of cows with suboptimal fertility and evaluate whether hormonal interventions would improve the reproductive efficiency of low-fertility cows. Cows in one herd were enrolled in the study at 50 days in milk (DIM) and classified as low or high fertility according to a proprietary Fertility Index. Weekly cohorts of cows were allocated to three treatments at 50 DIM: (1) high fertility and Presynch-Ovsynch (H-PO; n = 1036); (2) low fertility and Presynch-Ovsynch (L-PO; n = 665); (3) low fertility and Double-Ovsynch (L-DO; n = 645). The voluntary waiting period (VWP) was 65 and 50 DIM for first-lactation and second- and greater-lactation cows, respectively, regardless of the treatment group. For H-PO and L-PO treatments, Presynch-Ovsynch was initiated at 75 ± 3 DIM, and when cows were not inseminated early after detected estrus, timed artificial insemination (TAI) occurred at 113 ± 3 DIM. For cows assigned to the L-DO treatment, Double-Ovsynch was initiated at 53 ± 3 DIM, and when cows were not inseminated early after detected estrus, TAI occurred at 80 ± 3 DIM. All cows were eligible to be inseminated after the end of the VWP and during the entire presynchronization schemes. The Fertility Index successfully identified differences in pregnancy and culling outcomes regardless of the lactation number. Fewer cows (*p* < 0.01) predicted to have high compared with low fertility required a TAI for the first service, more (*p* < 0.01) were pregnant by 100 DIM, more (*p* < 0.01) were alive and pregnant by 250 DIM, fewer (*p* < 0.01) were culled by 250 DIM, and fewer tended (*p* = 0.08) to have pregnancy loss after first AI. Applying a fertility scheme (DO) to low-fertility cows failed to increase the proportion of cows pregnant by 250 DIM compared with low-fertility cows exposed to PO when cows were allowed to be inseminated at estrus during the entire presynchronization scheme. We conclude that submitting low-fertility cows to DO optimized the service rate for the first service; however, it did not increase pregnancy per AI compared with L-PO. Lastly, the Fertility Index successfully predicted differences in practical reproductive outcomes routinely monitored in dairy farms.

## Simple Summary

A proprietary Fertility Index was created by a machine-learning algorithm to identify potentially higher and lower subpopulations of cows in which the two subpopulations would differ in pregnancy per AI, speed of pregnancy creation, and herd survival. The Fertility Index successfully identified differences in pregnancy and culling outcomes. Submitting low-fertility cows to Double-Ovsynch reduced the days to first AI; however, it did not improve other reproductive outcomes compared with low-fertility cows exposed to Presynch-Ovsynch when cows were allowed to be inseminated at estrus during the entire presynchronization scheme.

## 1. Introduction

Improvements in reproduction and effective disease control in livestock production have been crucial to ensure global food security and sustainability [1,2]. In addition, advancements in herd health and the optimization of reproductive efficiency are essential for the profitability of cattle operations. In recent years, a variety of technologies and discoveries have played a significant role in advancing reproductive management in cattle farms [3].

Automated activity monitoring technology has been used for years to improve the efficiency and accuracy of estrus-detection programs. Recent studies have further explored the use of monitoring technology to optimize reproductive programs of lactating dairy cows, going beyond the use of automated alerts for insemination. Several research groups [4,5,6] have demonstrated it is possible to identify subpopulations of cows with subpar reproductive performance by using automated estrus alerts before the end of the voluntary waiting period (VWP). Using automated estrus alerts during the VWP to design targeted reproductive management programs aimed at optimizing artificial insemination (AI) based on estrus detection has been demonstrated [5,6].

Although different research groups have shown that automated estrus alerts may be useful to identify cows with suboptimal fertility, methods used to determine cows with or without alerts varied. For example, Laplacette et al. [6] disregarded estrus alerts that occurred before 15 days in milk (DIM) and those that were associated with pen moves. Borchardt et al. [4] evaluated the number of estrus alerts that occurred between 7 and 40 DIM. Although Gonzalez et al. [5] ignored estrus alerts before 10 DIM their heat-index level, which served as a proxy for estrus intensity, characterized if a cow had an estrus event during the VWP. In addition, a recent experiment conducted by Bretzinger et al. [7] reported discrepancies in the accuracy, sensitivity, and specificity across four automated activity monitoring systems in detecting anovulatory conditions within 60 DIM. Considering the inconsistency in characterizing estrus alerts during the VWP, a standardized method for each automated activity monitoring system is warranted to segregate cows into higher versus lower fertility.

Gonzalez et al. [5] hypothesized that a targeted reproductive program would reduce the need for exogenous hormones. In that study, cows that did not display estrus alerts before the end of the VWP were submitted to Double-Ovsynch (DO). It is likely that cows without estrus alerts may benefit from hormonal interventions; however, more research is needed to confirm this assumption. Furthermore, many herds in the U.S. rely on a prostaglandin-based presynchronization protocol (Presynch-Ovsynch, PO), whereas other herds employ GnRH–prostaglandin-based schemes such as DO. Results of a 27-study, 63-herd meta-analysis [8] confirmed that DO produced greater first service pregnancy per AI (P/AI) compared with PO in first-lactation cows (51.4 vs. 43.4%) but not in multiple-lactation cows (39.2 vs. 41.4%). Pregnancy losses during the second month of pregnancy did not differ between schemes. The researchers highlighted that the addition of GnRH included in the presynchronization portion of the DO scheme can induce ovulation in anovular cows before the initiation of the breeding Ovsynch, compared with a PO scheme, which is consistent with earlier studies [9,10].

It seems warranted to determine if a GnRH-based presynchronization protocol results in improved reproductive efficiency in cows expected to have suboptimal fertility. Therefore, the primary objective was to determine if a novel calculation created by a machine-learning algorithm, referred to as the Fertility Index, would correctly identify subpopulations of cows with lower reproductive efficiency in which the two subpopulations differed in pregnancy per AI or rate of pregnancy initiation when presynchronization protocols were similar. The secondary objective was to apply a fertility program, such as DO, to cows identified as having poorer reproductive performance based on the Fertility Index at a range in DIM consistent with industry standards, compared with cows identified as having low reproductive performance and submitted to a PO protocol. Specifically, we hypothesized that reproductive performance would be improved in high-fertility compared to low-fertility cows exposed to PO, and that exposing low-fertility cows to a fertility program, such as DO, would improve reproductive performance compared with low-fertility cows exposed to PO.

## 2. Materials and Methods

### 2.1. Cows, Enrollment Period, and Experimental Treatments

The experiment was conducted in a dairy located in the Southwest region of the United States (Kansas). Lactating cows were housed in a cross-ventilated free-stall barn. Cows were fed a total mixed diet calculated to meet nutrient requirements for gestating or lactating dairy cows producing 40 kg of 3.5% milk fat and provided water ad libitum [11]. At 240 days of gestation, nulliparous heifers were fitted with a neck-mounted behavior monitoring sensor tag (SenseHub Monitoring Neck Tag, Merck Animal Health, Rahway, NJ, USA) and were kept on the animals until they were culled from the herd. Cows were milked twice daily in a parallel parlor. Other procedures conformed to guidelines for the use of agricultural animals in research [12].

**Figure 1:**
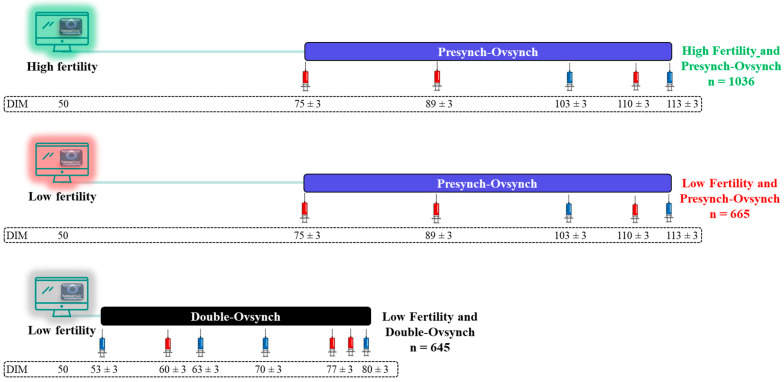
Experimental design scheme. Cows were classified as low or high fertility at 50 days in milk (DIM) according to the Fertility Index that was created by Merck Animal Health, Rahway, NJ, USA, based on a proprietary machine-learning algorithm. Weekly cohorts of cows were allocated to 3 treatments at 50 DIM: (1) high fertility and Presynch-Ovsynch (H-PO); (2) low fertility and Presynch-Ovsynch (L-PO); (3) low fertility and Double-Ovsynch (L-DO). All cows were fitted with a neck-mounted behavior-monitoring sensor tag to detect estrus alerts (SenseHub) and were eligible for insemination based on estrus alerts after the end of the VWP. Voluntary waiting period was 65 and 50 DIM for first-lactation and second- and greater-lactation cows, respectively, regardless of the treatment group. Initiation of the Presynch-Ovsynch and Double-Ovsynch protocols were 75 ± 3 and 53 ± 3 DIM, respectively, regardless of lactation number.

The enrollment period of the experiment was from March through September 2023. At 50 DIM, cows were classified as low or high fertility according to the Fertility Index created by Merck Animal Health, Rahway, NJ, USA based on a proprietary machine-learning algorithm. Briefly, the Fertility Index was generated based on days relative to calving, the lifecycle of production, and behaviors detected by the monitoring sensor tags, such as rumination, eating, and heavy breathing, among others, for feature engineering. Weekly cohorts of cows were allocated to 3 treatments at 50 DIM (Figure 1): (1) high fertility and Presynch-Ovsynch (H-PO; n = 1036); (2) low fertility and Presynch-Ovsynch (L-PO; n = 665); (3) low fertility and Double-Ovsynch (L-DO; n = 645). For low-fertility cows, allocation to treatment was based on the cow’s ear tag identification number. Cows with odd identification numbers were allocated to the L-PO treatment, whereas cows with even identification numbers were assigned to L-DO. The initiation of the Presynch-Ovsynch and Double-Ovsynch protocols occurred at 75 ± 3 and 53 ± 3 DIM, respectively, regardless of lactation number. The voluntary waiting period was 65 and 50 DIM for first-lactation and second- and greater-lactation cows, respectively, regardless of the treatment group.

For cows assigned to the H-PO and L-PO treatments, PO was initiated at 75 ± 3 DIM, and when cows were not inseminated early after detected estrus, timed artificial insemination (TAI) occurred at 113 ± 3 DIM. For cows assigned to the L-DO treatment, DO was initiated at 53 ± 3 DIM, and when cows were not inseminated early after detected estrus, TAI occurred at 80 ± 3 DIM.

All cows in each treatment were eligible to be inseminated after the end of the VWP. The detection of estrus was based on automated estrus alerts. Cows were inseminated once daily. When insemination occurred after detected estrus before the scheduled TAI, the PO or DO synchronization scheme was discontinued. The type of semen used at insemination was confounded with the lactation group. Sexed semen was used for all first inseminations in first-lactation cows; approximately 30% in second-lactation cows, and none in ≥third-lactation cows.

Reproductive hormones were administered i.m. and consisted of PGF2α (2 mL of Estrumate, 0.5 mg cloprostenol, Merck Animal Health, Rahway, NJ, USA) and GnRH (2 mL of Fertagyl, 100 mcg gonadorelin acetate, Merck Animal Health, Rahway, NJ, USA). Presynch-Ovsynch (PGF2α—14 d—PGF2α—14 d—GnRH—7 d—PGF2α—48 h—GnRH—24 h—TAI) and DO schemes (GnRH—7 d—PGF2α—3 d—GnRH—7 d—GnRH—7 d—PGF2α—24 h—PGF2α—24 h—GnRH—24 h—TAI) were consistent with current industry use [8,9,10].

### 2.2. Pregnancy Diagnosis and Resynchronization

Cows were submitted to pregnancy diagnosis at 38 ± 3 d after AI by transrectal ultrasonography, based on the detection of fluid and the presence of an embryo with a heartbeat. Nonpregnant cows were submitted to the P7G7 protocol [13] on the day of non-pregnancy diagnosis (i.e., cows received PGF2α followed in 7 d with GnRH). Cows not reinseminated, based on estrus alerts, were submitted to a TAI protocol: Cosynch-72 scheme (GnRH—7 d—PGF2α—72 h—GnRH +TAI). The same hormone doses and site of administration were used as described previously for the first inseminations. Cows diagnosed pregnant with a subsequent estrus alert were reinseminated and considered to have suffered pregnancy loss to the first insemination.

### 2.3. Temperature–Humidity Index

Relative humidity and temperature were collected from the nearest meteorological station, which was located approximately 30 km from the dairy farm to calculate the temperature–humidity index (THI) using the following equation [14]: T − (0.55 − [0.55 × RH/100] × [T − 58]); where T and RH are dry bulb temperature (°F).

### 2.4. Statistical Analyses

Dichotomous variables were analyzed by logistic regression (procedure GLIMMIX) in Statistical Analysis System (version 9.4, SAS Institute Inc., Cary, NC, USA). Options used in the model statement included LINK = LOGIT, DIST = BINOMIAL, and the least squares means option of ILINK and DIFF. The final models and their components included the fixed effects of treatment (n = 3), lactation group (first vs. second vs. ≥third), their interaction, and temperature–humidity index on the day of the first insemination. A power test (procedure POWER) was conducted in SAS (one-sided test) to determine the minimum sample size to avoid Type II errors based on percentage P/AI ranging from 34 to 40%. To achieve a 6-percentage point difference with the sample size of 1173 cows for H-PO and 655 cows in either L-PO or L-DO treatment, the power of 0.80 was achieved with an alpha = 0.05. Time to insemination, culling, or pregnancy was analyzed by Cox proportional hazard regression (procedure PHREG) in SAS. The final models applied the same model as described previously for the dichotomous variables. Survival analysis for time to events was performed using the LIFETEST procedure in SAS. The analyses that explored the likelihood or time to pregnancy up to 250 DIM included the latest DIM to conception to account for pregnancy losses. The endpoint of the analysis was pregnancy. Therefore, if a cow was culled after pregnancy confirmation, cows were deemed pregnant and not censored in the survival curve. Conversely, in the analyses that evaluated the likelihood and time to survival and pregnancy by 250 DIM, cows were deemed not pregnant if culled in the first 250 DIM, regardless of pregnancy status. Statistical significance of effects was set as *p* ≤ 0.05, with tendencies as 0.05 < *p* ≤ 0.10.

## 3. Results

### 3.1. First Insemination and Pregnancy at 100 DIM

Neither treatment nor the interaction between treatment and lactation group (*p* ≥ 0.28) was associated with the percentage of cows submitted to the first service (Table 1).

**Figure 2:**
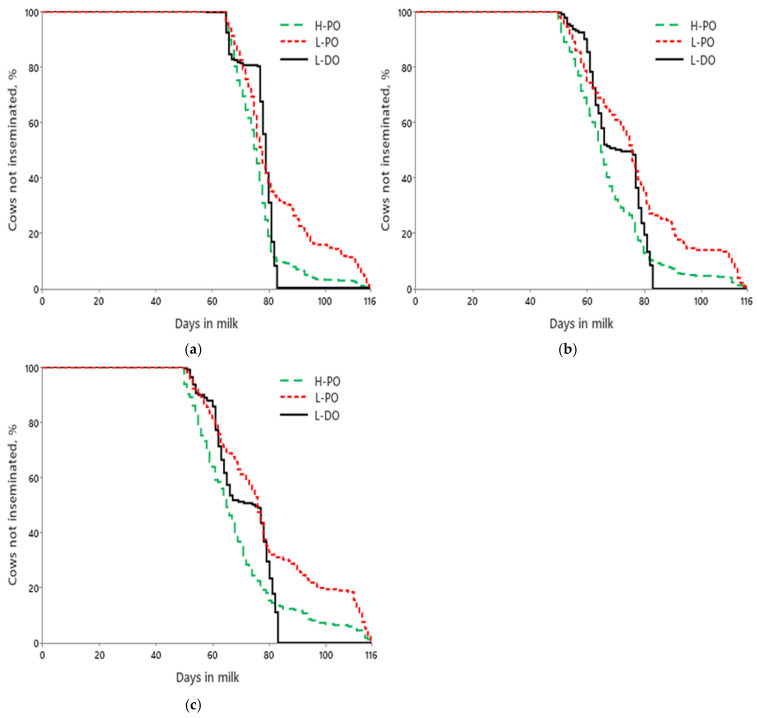
Effects of treatment on days to first breeding first-(**a**), second-(**b**), and ≥third-lactation (**c**) cows. (**a**) Mean days to first breeding were 76.3 ± 0.4, 82.7 ± 0.9, and 77.1 ± 0.3 d for H-PO, L-PO, and L-DO cows, respectively (*p* < 0.01). Median days to first AI were 76, 78, and 79 d for H-PO, L-PO, and L-DO cows, respectively; (**b**) Mean days to pregnancy were 67.8 ± 0.8, 77.0 ± 1.4, and 70.5 ± 0.7 d for H-PO, L-PO, and L-DO cows, respectively (*p* < 0.01). Median days to first AI were 65, 76, and 72 d for H-PO, L-PO, and L-DO cows, respectively; (**c**) Mean days to pregnancy were 68.6 ± 1.0, 79.1 ± 1.3, and 70.6 ± 0.8 d for H-PO, L-PO, and L-DO cows, respectively (*p* < 0.01). Median days to first AI were 65, 76, and 75 d for H-PO, L-PO, and L-DO cows, respectively.

Treatment, lactation group, and THI affected (*p* < 0.01) DIM at first service. High fertility (70.7 ± 0.4 d) and L-PO (79.0 ± 0.5 d) cows had the least and greatest average DIM at first service, respectively, whereas L-DO (72.8 ± 0.5 d) was intermediate. The interaction between treatment and lactation group did not (*p* = 0.11) affect DIM to the first AI. Hazard of first insemination was affected (*p* ≤ 0.04) by treatment, lactation group, THI, and the interaction between treatment and lactation group. Across lactation groups, H-PO cows were inseminated at a faster rate, L-PO at a slower rate, and L-DO was intermediate between the other treatments (Figure 2a–c). Low-fertility cows exposed to PO had a reduced (*p* < 0.02) hazard of insemination than L-DO across all lactations.

When only pregnant cows were included in the Cox proportional hazard for the first service, first-lactation H-PO were inseminated at a faster (*p* < 0.01) rate than first-lactation L-PO, whereas L-DO did not differ (*p* ≥ 0.11) with the other treatments. For second-lactation cows, H-PO and L-DO were inseminated at a faster (*p* < 0.01) rate than L-PO, and no differences (*p* = 0.25) were detected between H-PO and L-DO. For ≥third-lactation cows, H-PO and L-DO were inseminated at a faster (*p* < 0.01) rate than L-PO, and H-PO tended (*p* = 0.09) to differ from L-DO.

Treatment and the interaction between treatment and lactation group affected (*p* < 0.01) the percentage of cows submitted to TAI (Table 1 and Table 2).

Lactation group, THI, and the interaction between treatment and lactation group affected (*p* < 0.02) the first service P/AI (Table 1). No differences were detected in P/AI between the treatments for first-lactation and second-lactation cows (Table 3). For ≥third-lactation cows; however, H-PO cows had (*p* < 0.01) and tended (*p* = 0.08) to have greater P/AI than L-DO and L-PO, respectively (Table 3). No differences (*p* = 0.11) were detected between L-DO and L-PO.

For cows that became pregnant in the first service, treatment tended to affect pregnancy loss (Table 1). High-fertility cows had (*p* = 0.03) and tended (*p* = 0.06) to have fewer pregnancy losses than L-PO and L-DO, respectively. No differences (*p* = 0.76) were detected between L-PO and L-DO. No other variables were associated with pregnancy loss.

For cows that became pregnant in the lactation, treatment, lactation group, its interaction and THI were associated (*p* < 0.01) with pregnancy by 100 DIM (Table 1). For first-lactation cows, high fertility had (*p* = 0.02) and tended (*p* = 0.08) to have a greater percentage of cows pregnant by 100 DIM than L-DO and L-PO, respectively. For second-lactation cows, L-PO had a decreased (*p* ≤ 0.02) percentage of cows pregnant by 100 DIM compared to H-PO and L-DO. For ≥third-lactation cows, H-PO had an increased (*p* < 0.01) percentage of pregnant cows compared to L-PO and L-DO (Table 3).

### 3.2. Likelihood and Hazard of Pregnancy and Culling up to 250 DIM

Treatment and lactation group affected (*p* < 0.01) and THI tended (*p* = 0.08) to affect the likelihood of cows becoming pregnant by 250 DIM (Table 1). High-fertility cows were more likely to become pregnant than L-PO and L-DO cows. The interaction between treatment and lactation group was not (*p* = 0.19) associated with pregnancy outcome by 250 DIM. In contrast, hazard of pregnancy was affected (*p* = 0.03) by the interaction treatment and lactation group (Table 4).

**Figure 3:**
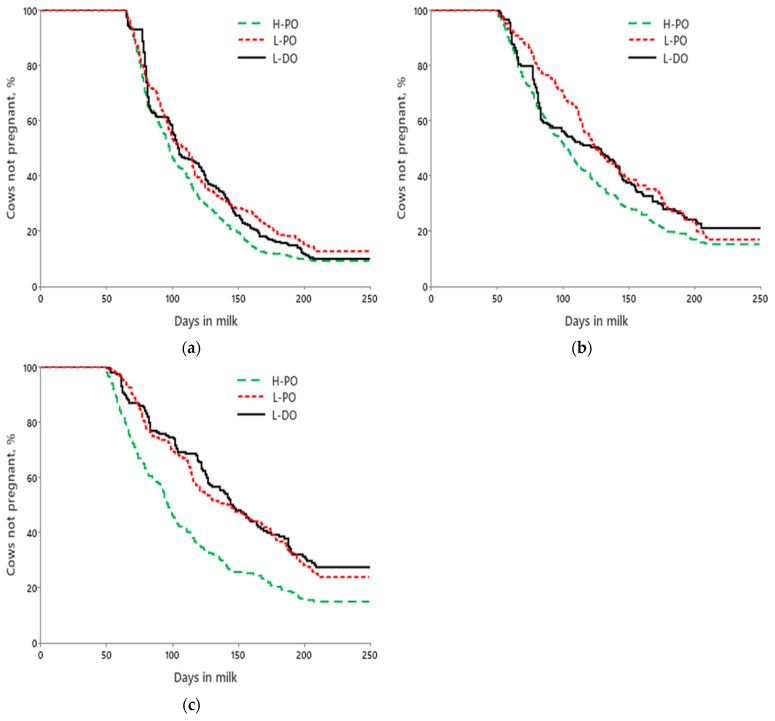
Effects of treatment on days to pregnancy up to 250 DIM for first-(**a**), second-(**b**), and ≥third-lactation (**c**) cows. (**a**) Mean days to pregnancy were 112.2 ± 2.1, 123.2 ± 3.0, and 120.8 ± 2.8 d for H-PO, L-PO, and L-DO cows, respectively (*p* < 0.01); (**b**) Mean days to pregnancy were 118.4 ± 3.0, 136.5 ± 4.2, and 128.1 ± 4.4 d for H-PO, L-PO, and L-DO cows, respectively (*p* < 0.01); (**c**) Mean days to pregnancy were 114.3 ± 3.5, 143.9 ± 3.9, and 145.8 ± 4.2 d for H-PO, L-PO, and L-DO cows, respectively (*p* < 0.01).

For first-lactation cows, the median days to pregnancy to 250 DIM were 98, 109, and 104 for H-PO, L-PO, and L-DO (Figure 3a), respectively. For second-lactation cows, the median days to pregnancy were 103, 125, and 127 for H-PO, L-PO, and L-DO (Figure 3b), respectively. For ≥third-lactation cows, the median days to pregnancy were 97, 142, and 144 for H-PO, L-PO, and L-DO (Figure 3c), respectively.

High-fertility cows were less likely (*p* ≤ 0.02) to be culled by 250 DIM than L-PO and L-DO (Table 1). First-lactation cows were less likely to be culled than second- and ≥third-lactation cows. Second-lactation cows tended (*p* = 0.10) to have reduced culling compared to ≥third-lactation cows. No interaction was detected (*p* = 0.37) between treatment and lactation group.

The likelihood of cows being alive and pregnant by 250 DIM was affected (*p* ≤ 0.05) by treatment, lactation group, and THI. Both low-fertility treatments (L-PO and L-DO) had fewer pregnant and live cows at 250 DIM than H-PO. No interaction was detected (*p* = 0.35) between treatment and lactation group.

**Figure 4:**
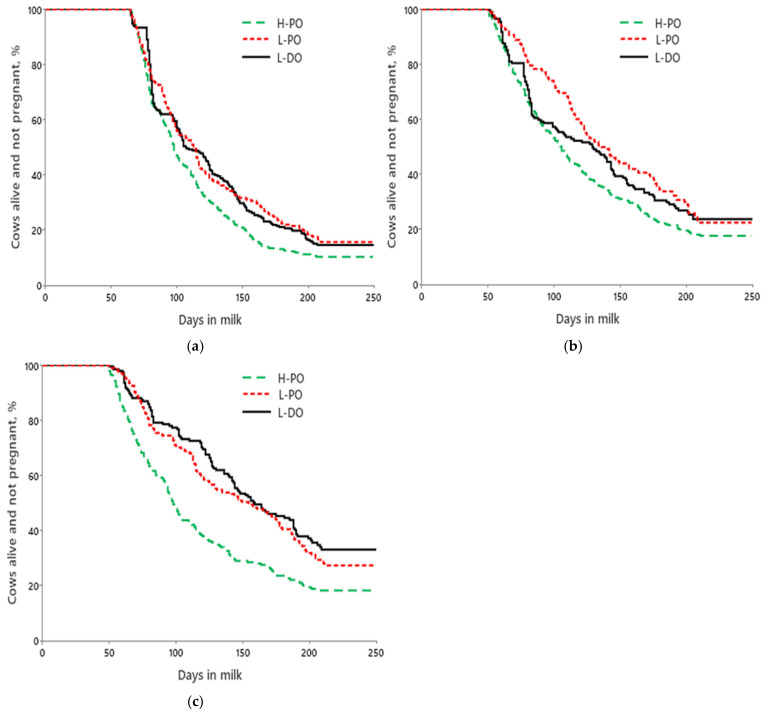
Effects of treatment on survival and days to pregnancy up to 250 DIM for first-(**a**), second-(**b**), and ≥third-lactation (**c**) cows. (**a**) Mean days to pregnancy were 113.5 ± 2.1, 127.2 ± 3.2, and 125.2 ± 3.0 d for H-PO, L-PO, and L-DO cows, respectively (*p* < 0.01); (**b**) Mean days to pregnancy were 121.4 ± 3.1, 143.2 ± 4.4, and 130.3 ± 4.5 d for H-PO, L-PO, and L-DO cows, respectively (*p* < 0.01); (**c**) Mean days to pregnancy were 117.9 ± 3.6, 147.7 ± 3.9, and 152.3 ± 4.2 d for H-PO, L-PO, and L-DO cows, respectively (*p* < 0.01).

The hazard of pregnancy and survival by 250 DIM was affected (*p* ≤ 0.05) by treatment, lactation group, their interaction, and THI. High-fertility cows had a greater hazard of pregnancy by 250 DIM than both low-fertility treatments (L-PO and L-DO) across all lactation groups (Table 4). For first-lactation cows that were not culled by 250 DIM, the median days to pregnancy were 98, 113, and 107 for H-PO, L-PO, and L-DO (Figure 4a), respectively. For second-lactation cows that were not culled by 250 DIM, the median days to pregnancy were 105, 136, and 129 for H-PO, L-PO, and L-DO (Figure 4b), respectively. For ≥third-lactation cows, the median days to pregnancy were 98, 155, and 159 for H-PO, L-PO, and L-DO (Figure 4c), respectively.

## 4. Discussion

With the advent of automated activity-monitoring technology and its increased adoption, various researchers have undertaken studies to elucidate associations between cow behavior and reproductive efficiency [15,16]. In fact, monitoring technology has assisted researchers to further understand the intricacies of reproductive biology, management, and reproductive efficiency. Like work conducted with biomarkers [17,18,19], sensor technology is promising in its potential to help identify subpopulations of cows with suboptimal performance. Nevertheless, further research is needed to focus on predictive models rather than solely exploring associations. In addition, predictive studies should be complemented by research aimed at exploring and implementing potential solutions that may overcome shortcomings in the fertility of specific subgroups of cows. The current study was designed to proactively identify cows with suboptimal performance and attempt to improve their reproductive outcomes by utilizing a presynchronization program before the first AI, tailored to induce ovulation with additional injections of GnRH, regardless of cyclic status.

The Fertility Index successfully identified differences in pregnancy and culling outcomes. In general, regardless of the lactation number, fewer cows predicted to have high fertility compared with low fertility required TAI, more were pregnant by 100 DIM, more were alive and pregnant by 250 DIM, fewer were culled by 250 DIM, and fewer tended to have pregnancy loss after the first AI. More rapid pregnancy creation in the high-fertility cows occurred despite no overall treatment differences in P/AI at the first service except for ≥third-lactation cows. In the latter lactation group, P/AI in H-PO was greater than in L-DO cows and tended to be greater than in L-PO cows. These differences also occurred despite differences in DIM at the first AI between high- and low-fertility cows because H-PO cows were inseminated at a faster rate than L-DO cows. Therefore, one component of high fertility is the ability of cows to express earlier postpartum fertile periods of estrus plus a tendency for less pregnancy loss after those pregnancies were created.

Low-fertility cows treated with PO had the greatest DIM at the first service. It is possible that the extended DIM at the first service resulted in greater P/AI because extending DIM at AI is associated with greater P/AI [20,21]. In addition, the potential benefit of exposing L-DO cows to a fertility program may have been diminished by expediting the first service. We speculate that this is one of the reasons we did not observe major differences between the PO and DO protocols, as previously reported [8]. Despite the advantage of a scheduled earlier first AI in L-DO cows, H-PO cows (first- and ≥third-lactation cows) were more likely to be pregnant by 100 DIM than L-DO cows. Furthermore, low-fertility issues cannot be overcome by inseminating earlier after calving, at least when applying the DO scheme. In other words, even though the service rate was optimized with DO, it did not translate into differences in the percentage of cows pregnant by 100 DIM in low-fertility cows. Using DO in low-fertility cows seemed to expedite pregnancy creation in second-lactation cows, but that difference disappeared later as percentage pregnant in L-PO cows caught up. Double-Ovsynch failed to improve pregnancy creation in ≥third-lactation cows consistent with other results [8]. The DO scheme was chosen because of its ability to improve P/AI in first-lactation cows compared with prostaglandin presynchronization programs [8]. The DO scheme can reduce anovulation before insemination [9,10] because of multiple pre-breeding exposures to GnRH and GnRH-induced LH release [22], and its relationship to ovarian steroids and subsequent ovulation is well documented [23].

Allowing cows to be inseminated after the detection of estrus during the DO program may be another reason why we did not observe differences in P/AI at the first service between DO and PO. We could not find a study in which AI was conducted after estrus during the entire DO scheme as in L-DO treatment. Early insemination conducted after estrus in four herds, during which cows were exposed to PO and PG-3-G (PG—3 d—GnRH) before Ovsynch, resulted in lower P/AI at first AI during all seasons of the year compared with cows receiving TAI, regardless of the presynchronization scheme [23]. When cows were submitted to AI after estrus when exposed to various presynchronization schemes in six studies, but not during the entire scheme as in the present study, those cows receiving only the TAI had a greater P/AI than cows inseminated at estrus [24].

Interpreting our PO results, considering a meta-analysis in which cows with 100% TAI after completing a PO scheme had more P/AI at first AI than cows inseminated after PO, which included AI at detected estrus after the second PGF2α injection of the PO scheme [25], should be clear. The latter results were achieved in herds with DIM at first AI ranging from 59 to 81 DIM. Those conditions were quite different from how cows were managed in the PO treatments herein, in which cows were eligible for AI anytime after the end of the VWP during the entire PO scheme, and TAI was not conducted until 113 ± 3 DIM.

The effectiveness of the developed Fertility Index is validated by several outcomes assessed in this first study. Even though PO cows initiated the program later, after the end of the VWP, resulting in the first TAI at 113 ± 3 DIM for cows not identified in estrus and previously inseminated, L-DO (TAI at 80 ± 3 DIM) had a worse overall performance than H-PO in all lactation groups. Pregnancy loss after the first AI tended to be higher, more culling occurred, and fewer low-fertility cows were alive and pregnant by 250 DIM compared with high-fertility cows. These results further validate the ability of the Fertility Index to predict outcomes when cows are inseminated at similar or different DIM. Given the effectiveness of the Fertility Index, other synchronization programs should be evaluated in low-fertility cows to determine if reproductive efficiency in these cows can be improved.

Absolute pregnancy outcomes should be interpreted and viewed in the context of both season and semen types used in this study. Treatments were conducted during the months of March through September when seasonal effects on fertility were clearly compromised [26]. Furthermore, results of P/AI in first-lactation cows were achieved with only sexed semen, whereas sexed semen was used limitedly in second-lactation cows and none in ≥third-lactation cows. Given these limitations, the Fertility Index was robust and accurate in identifying cows with different pregnancy outcomes. Further work is warranted to apply the Fertility Index in other herds when cows are first inseminated at industry-standard DIM (range of 30 to 90; mean = 56 ± 0.6 DIM; [27,28]) practiced in the U.S.

## 5. Conclusions

In the current study, DO did not increase P/AI for the first breeding of low-fertility cows compared with PO. Double-Ovsynch optimized service rate for the first service as differences were observed in the hazard of the first insemination. High-fertility cows were inseminated at a faster rate, L-PO at a slower rate, and L-DO was intermediate between the other treatments. Nevertheless, the GnRH-based presynchronization strategy did not result in a greater proportion of cows pregnant by 250 DIM for low-fertility cows. Overall, applying a fertility scheme (DO) to low-fertility cows failed to improve their main reproductive outcomes compared with low-fertility cows exposed to PO, when cows were allowed to be inseminated at estrus during the entire presynchronization scheme. The Fertility Index successfully identified two pre-breeding fertility subpopulations of dairy cows substantiated by resulting pregnancy outcomes. Further research is needed to investigate whether other presynchronization schemes can restore the reproductive efficiency of low-fertility cows.

## 6. Patents

A patent application for the Fertility Index has been filed by L.G.D.M., K.W., G.F., and T.B. The pending patent application is US Patent Application No. 18/741,859.

## Figures and Tables

**Table 1:** Effect of treatment on reproductive traits and culling (LSM ± SEM).

	Treatment (T) ^1^			*p*-Value		
Item	H-PO	L-PO	L-DO	T	Lactation Group	Interaction
Number of cows	1036	665	645	-	-	-
Submitted to first AI, %	97.5 ± 0.5	97.6 ± 0.6	97.5 ± 0.7	0.99	0.28	0.94
Submitted to first service timed AI, %	3.3 ± 0.6	13.6 ± 1.4	60.6 ± 2.1	<0.01	0.22	<0.01
Pregnancy per AI for first service, %	38.1 ± 1.6	34.9 ± 2.0	33.8 ± 2.0	0.19	<0.01	0.02
Pregnancy loss from first service, %	1.6 ± 0.7	5.0 ± 1.5	4.4 ± 1.4	0.08	0.16	0.81
Pregnant by 100 DIM, %	51.1 ± 1.6	35.7 ± 2.0	37.3 ± 2.0	<0.01	<0.01	<0.01
Pregnant by 250 DIM, %	84.4 ± 1.2	78.8 ± 1.7	76.7 ± 1.8	<0.01	<0.01	0.19
Alive and pregnant by 250 DIM, %	81.9 ± 1.2	74.1 ± 1.8	72.2 ± 1.9	<0.01	<0.01	0.35
Culled by 250 DIM, %	8.2 ± 0.9	11.8 ± 1.3	13.3 ± 1.4	<0.01	<0.01	0.37

^1^ High fertility and Presynch-Ovsynch (H-PO); low fertility and Presynch-Ovsynch (L-PO); and low fertility and Double-Ovsynch (L-DO).

**Table 2:** Proportion of cows submitted to timed AI (TAI) at first service based on treatment ^1^ and lactation group.

Item	LSM ± SEM ^2^	AOR ^3^ (95% CI)	*p*-Value
Submitted to TAI: first lactation			
H-PO	2.5 ± 0.7	1.00	-
L-PO	11.4 ± 1.9	5.0 (2.5–10.2)	<0.01
L-DO	80.2 ± 2.4	159.2 (81.6–311.0)	<0.01
Submitted to TAI: second lactation			
H-PO	3.3 ± 1.0	1.00	-
L-PO	13.0 ± 2.6	4.3 (2.0–9.1)	<0.01
L-DO	48.8 ± 3.9	27.5 (14.0–54.0)	<0.01
Submitted to TAI: ≥third lactation			
H-PO	4.2 ± 1.3	1.00	-
L-PO	17.0 ± 2.5	4.7 (2.3–9.7)	<0.01
L-DO	48.6 ± 3.7	21.7 (10.8–43.7)	<0.01

^1^ High fertility and Presynch-Ovsynch (H-PO); low fertility and Presynch-Ovsynch (L-PO); and low fertility and Double-Ovsynch (L-DO). ^2^ Least squares mean ± standard error. ^3^ Adjusted odds ratios and 95% confidence intervals.

**Table 3:** Pregnancy per AI (P/AI) and percentage of cows that became pregnant by 100 days in milk, according to treatment ^1^ and lactation group.

Item	LSM ± SEM ^2^	AOR ^3^ (95% CI)	*p*-Value
P/AI: first lactation			
H-PO	41.4 ± 2.4	1.00	-
L-PO	41.5 ± 3.0	1.00 (0.74–1.37)	0.98
L-DO	39.0 ± 2.9	0.91 (0.67–1.24)	0.54
P/AI: second lactation			
H-PO	34.7 ± 2.6	1.00	-
L-PO	32.7 ± 3.7	0.92 (0.61–1.37)	0.67
L-DO	40.3 ± 3.8	1.27 (0.87–1.87)	0.22
P/AI: ≥third lactation			
H-PO	38.5 ± 3.1	1.00	-
L-PO	30.9 ± 3.2	0.71 (0.48–1.05)	0.09
L-DO	23.6 ± 3.2	0.49 (0.32–0.76)	<0.01
Pregnant by 100 DIM: first lactation			
H-PO	53.6 ± 2.4	1.00	-
L-PO	46.7 ± 3.1	0.76 (0.56–1.03)	0.08
L-DO	44.8 ± 3.0	0.70 (0.52–0.95)	0.02
Pregnant by 100 DIM: second lactation			
H-PO	47.5 ± 2.8	1.00	-
L-PO	30.8 ± 3.7	0.49 (0.33–0.73)	<0.01
L-DO	43.4 ± 3.9	0.85 (0.58–1.23)	0.38
Pregnant by 100 DIM: ≥third lactation			
H-PO	52.1 ± 3.2	1.00	-
L-PO	30.4 ± 3.2	0.40 (0.27–0.59)	<0.01
L-DO	25.3 ± 3.2	0.31 (0.20–0.47)	<0.01

^1^ High fertility and Presynch-Ovsynch (H-PO); low fertility and Presynch-Ovsynch (L-PO); and low fertility and Double-Ovsynch (L-DO). ^2^ Least squares mean ± standard error. ^3^ Adjusted odds ratios and 95% confidence intervals.

**Table 4:** Effect of treatment on hazard of survival and pregnancy by 250 DIM according to treatment ^1^ and lactation group.

Item	AHR ^2^ (95% CI)	*p*-Value
Pregnancy by 250 DIM: first lactation		
H-PO	1.00	-
L-PO	0.82 (0.70–0.97)	0.02
L-DO	0.85 (0.73–1.00)	0.05
Pregnancy by 250 DIM: second lactation		
H-PO	1.00	-
L-PO	0.77 (0.62–0.94)	0.01
L-DO	0.79 (0.64–0.98)	0.03
Pregnancy by 250 DIM: ≥third lactation		
H-PO	1.00	-
L-PO	0.61 (0.49–0.75)	<0.01
L-DO	0.56 (0.44–0.70)	<0.01
Alive and pregnancy by 250 DIM: first lactation		
H-PO	1.00	-
L-PO	0.78 (0.66–0.92)	<0.01
L-DO	0.79 (0.67–0.93)	<0.01
Alive and pregnancy by 250 DIM: second lactation		
H-PO	1.00	-
L-PO	0.71 (0.57–0.88)	<0.01
L-DO	0.81 (0.65–1.00)	0.05
Alive and pregnancy by 250 DIM: ≥third lactation		
H-PO	1.00	-
L-PO	0.62 (0.50–0.76)	<0.01
L-DO	0.53 (0.42–0.67)	<0.01

^1^ High fertility and Presynch-Ovsynch (H-PO); low fertility and Presynch-Ovsynch (L-PO); and low fertility and Double-Ovsynch (L-DO). ^2^ Adjusted hazard ratios and 95% confidence intervals.

## Data Availability

The original contributions presented in the study are included in the article, further inquiries can be directed to the corresponding author.
